# Supplementary material for: Analysis and design of single-cell experiments to harvest fluctuation information while rejecting measurement noise
Source: Front Cell Dev Biol. 2023 May 26;11:1133994. doi: 10.3389/fcell.2023.1133994 (PMC10250612; doi:10.3389/fcell.2023.1133994)
Supplement: Supplementary file 1 [file DataSheet2.PDF]

Below is the operating system and hardware specifications used to run codes in this manuscript.

```
******************************
PYTHON Image Processing Codes

Linux ens13487 4.15.0-166-generic #174-Ubuntu SMP Wed Dec 8 19:07:44
UTC 2021 x86_64 x86_64 x86_64 GNU/Linux

Architecture:        x86_64
CPU op-mode(s):      32-bit, 64-bit
Byte Order:          Little Endian
CPU(s):              32
On-line CPU(s) list: 0-31
Thread(s) per core:  2
Core(s) per socket:  16
Socket(s):           1
NUMA node(s):        2s
Vendor ID:           AuthenticAMD
CPU family:          23
Model:               8
Model name:          AMD Ryzen Threadripper 2950X 16-Core Processor
Stepping:            2
CPU MHz:             1893.365
CPU max MHz:         3500.0000
CPU min MHz:         2200.0000
BogoMIPS:            6986.34
Virtualization:      AMD-V
L1d cache:           32K
L1i cache:           64K
L2 cache:            512K
L3 cache:            8192K
NUMA node0 CPU(s):   0-15
NUMA node1 CPU(s):   16-31
Flags:               fpu vme de pse tsc msr pae mce cx8 apic sep mtrr
pge mca cmov pat pse36 clflush mmx fxsr sse sse2 ht syscall nx mmxext
fxsr_opt pdpe1gb rdtscp lm constant_tsc rep_good nopl nonstop_tsc
cpuid extd_apicid amd_dcm aperfmperf pni pclmulqdq monitor ssse3 fma
cx16 sse4_1 sse4_2 movbe popcnt aes xsave avx f16c rdrand lahf_lm
cmp_legacy svm extapic cr8_legacy abm sse4a misalignsse 3dnowprefetch
osvw skinit wdt tce topoext perfctr_core perfctr_nb bpext perfctr_llc
mwaitx cpb hw_pstate sme ssbd ibpb vmmcall fsgsbase bmi1 avx2 smep
bmi2 rdseed adx smap clflushopt sha_ni xsaveopt xsavec xgetbv1 xsaves
clzero irperf xsaveerptr arat npt lbrv svm_lock nrip_save tsc_scale
vmcb_clean flushbyasid decodeassists pausefilter pfthreshold avic
v_vmsave_vmload vgif overflow_recov succor smca

00:00.0 Host bridge: Advanced Micro Devices, Inc. [AMD] Family 17h
(Models 00h-0fh) Root Complex
00:00.2 IOMMU: Advanced Micro Devices, Inc. [AMD] Family 17h (Models
```

```
00h-0fh) I/O Memory Management Unit
00:01.0 Host bridge: Advanced Micro Devices, Inc. [AMD] Family 17h
(Models 00h-0fh) PCIe Dummy Host Bridge
00:01.1 PCI bridge: Advanced Micro Devices, Inc. [AMD] Family 17h
(Models 00h-0fh) PCIe GPP Bridge
00:02.0 Host bridge: Advanced Micro Devices, Inc. [AMD] Family 17h
(Models 00h-0fh) PCIe Dummy Host Bridge
00:03.0 Host bridge: Advanced Micro Devices, Inc. [AMD] Family 17h
(Models 00h-0fh) PCIe Dummy Host Bridge
00:03.1 PCI bridge: Advanced Micro Devices, Inc. [AMD] Family 17h
(Models 00h-0fh) PCIe GPP Bridge
00:04.0 Host bridge: Advanced Micro Devices, Inc. [AMD] Family 17h
(Models 00h-0fh) PCIe Dummy Host Bridge
00:07.0 Host bridge: Advanced Micro Devices, Inc. [AMD] Family 17h
(Models 00h-0fh) PCIe Dummy Host Bridge
00:07.1 PCI bridge: Advanced Micro Devices, Inc. [AMD] Family 17h
(Models 00h-0fh) Internal PCIe GPP Bridge 0 to Bus B
00:08.0 Host bridge: Advanced Micro Devices, Inc. [AMD] Family 17h
(Models 00h-0fh) PCIe Dummy Host Bridge
00:08.1 PCI bridge: Advanced Micro Devices, Inc. [AMD] Family 17h
(Models 00h-0fh) Internal PCIe GPP Bridge 0 to Bus B
00:14.0 SMBus: Advanced Micro Devices, Inc. [AMD] FCH SMBus Controller
(rev 59)
00:14.3 ISA bridge: Advanced Micro Devices, Inc. [AMD] FCH LPC Bridge
(rev 51)
00:18.0 Host bridge: Advanced Micro Devices, Inc. [AMD] Family 17h
(Models 00h-0fh) Data Fabric: Device 18h; Function 0
00:18.1 Host bridge: Advanced Micro Devices, Inc. [AMD] Family 17h
(Models 00h-0fh) Data Fabric: Device 18h; Function 1
00:18.2 Host bridge: Advanced Micro Devices, Inc. [AMD] Family 17h
(Models 00h-0fh) Data Fabric: Device 18h; Function 2
00:18.3 Host bridge: Advanced Micro Devices, Inc. [AMD] Family 17h
(Models 00h-0fh) Data Fabric: Device 18h; Function 3
00:18.4 Host bridge: Advanced Micro Devices, Inc. [AMD] Family 17h
(Models 00h-0fh) Data Fabric: Device 18h; Function 4
00:18.5 Host bridge: Advanced Micro Devices, Inc. [AMD] Family 17h
(Models 00h-0fh) Data Fabric: Device 18h; Function 5
00:18.6 Host bridge: Advanced Micro Devices, Inc. [AMD] Family 17h
(Models 00h-0fh) Data Fabric: Device 18h; Function 6
00:18.7 Host bridge: Advanced Micro Devices, Inc. [AMD] Family 17h
(Models 00h-0fh) Data Fabric: Device 18h; Function 7
00:19.0 Host bridge: Advanced Micro Devices, Inc. [AMD] Family 17h
(Models 00h-0fh) Data Fabric: Device 18h; Function 0
00:19.1 Host bridge: Advanced Micro Devices, Inc. [AMD] Family 17h
(Models 00h-0fh) Data Fabric: Device 18h; Function 1
00:19.2 Host bridge: Advanced Micro Devices, Inc. [AMD] Family 17h
(Models 00h-0fh) Data Fabric: Device 18h; Function 2
00:19.3 Host bridge: Advanced Micro Devices, Inc. [AMD] Family 17h
(Models 00h-0fh) Data Fabric: Device 18h; Function 3
00:19.4 Host bridge: Advanced Micro Devices, Inc. [AMD] Family 17h
```

```
(Models 00h-0fh) Data Fabric: Device 18h; Function 4
00:19.5 Host bridge: Advanced Micro Devices, Inc. [AMD] Family 17h
(Models 00h-0fh) Data Fabric: Device 18h; Function 5
00:19.6 Host bridge: Advanced Micro Devices, Inc. [AMD] Family 17h
(Models 00h-0fh) Data Fabric: Device 18h; Function 6
00:19.7 Host bridge: Advanced Micro Devices, Inc. [AMD] Family 17h
(Models 00h-0fh) Data Fabric: Device 18h; Function 7
01:00.0 USB controller: Advanced Micro Devices, Inc. [AMD] X399 Series
Chipset USB 3.1 xHCI Controller (rev 02)
01:00.1 SATA controller: Advanced Micro Devices, Inc. [AMD] X399
Series Chipset SATA Controller (rev 02)
01:00.2 PCI bridge: Advanced Micro Devices, Inc. [AMD] X399 Series
Chipset PCIe Bridge (rev 02)
02:00.0 PCI bridge: Advanced Micro Devices, Inc. [AMD] 300 Series
Chipset PCIe Port (rev 02)
02:01.0 PCI bridge: Advanced Micro Devices, Inc. [AMD] 300 Series
Chipset PCIe Port (rev 02)
02:02.0 PCI bridge: Advanced Micro Devices, Inc. [AMD] 300 Series
Chipset PCIe Port (rev 02)
02:03.0 PCI bridge: Advanced Micro Devices, Inc. [AMD] 300 Series
Chipset PCIe Port (rev 02)
02:04.0 PCI bridge: Advanced Micro Devices, Inc. [AMD] 300 Series
Chipset PCIe Port (rev 02)
04:00.0 Ethernet controller: Intel Corporation I211 Gigabit Network
Connection (rev 03)
05:00.0 Network controller: Intel Corporation Wireless 8265 / 8275
(rev 78)
06:00.0 Ethernet controller: Intel Corporation I211 Gigabit Network
Connection (rev 03)
08:00.0 VGA compatible controller: NVIDIA Corporation Device 1f02 (rev
a1)
08:00.1 Audio device: NVIDIA Corporation Device 10f9 (rev a1)
08:00.2 USB controller: NVIDIA Corporation Device 1ada (rev a1)
08:00.3 Serial bus controller [0c80]: NVIDIA Corporation Device 1adb
(rev a1)
09:00.0 Non-Essential Instrumentation [1300]: Advanced Micro Devices,
Inc. [AMD] Device 145a
09:00.2 Encryption controller: Advanced Micro Devices, Inc. [AMD]
Family 17h (Models 00h-0fh) Platform Security Processor
09:00.3 USB controller: Advanced Micro Devices, Inc. [AMD] USB 3.0
Host controller
0a:00.0 Non-Essential Instrumentation [1300]: Advanced Micro Devices,
Inc. [AMD] Device 1455
0a:00.2 SATA controller: Advanced Micro Devices, Inc. [AMD] FCH SATA
Controller [AHCI mode] (rev 51)
0a:00.3 Audio device: Advanced Micro Devices, Inc. [AMD] Family 17h
(Models 00h-0fh) HD Audio Controller
40:00.0 Host bridge: Advanced Micro Devices, Inc. [AMD] Family 17h
(Models 00h-0fh) Root Complex
40:00.2 IOMMU: Advanced Micro Devices, Inc. [AMD] Family 17h (Models
```

```
00h-0fh) I/O Memory Management Unit
40:01.0 Host bridge: Advanced Micro Devices, Inc. [AMD] Family 17h
(Models 00h-0fh) PCIe Dummy Host Bridge
40:02.0 Host bridge: Advanced Micro Devices, Inc. [AMD] Family 17h
(Models 00h-0fh) PCIe Dummy Host Bridge
40:03.0 Host bridge: Advanced Micro Devices, Inc. [AMD] Family 17h
(Models 00h-0fh) PCIe Dummy Host Bridge
40:03.1 PCI bridge: Advanced Micro Devices, Inc. [AMD] Family 17h
(Models 00h-0fh) PCIe GPP Bridge
40:04.0 Host bridge: Advanced Micro Devices, Inc. [AMD] Family 17h
(Models 00h-0fh) PCIe Dummy Host Bridge
40:07.0 Host bridge: Advanced Micro Devices, Inc. [AMD] Family 17h
(Models 00h-0fh) PCIe Dummy Host Bridge
40:07.1 PCI bridge: Advanced Micro Devices, Inc. [AMD] Family 17h
(Models 00h-0fh) Internal PCIe GPP Bridge 0 to Bus B
40:08.0 Host bridge: Advanced Micro Devices, Inc. [AMD] Family 17h
(Models 00h-0fh) PCIe Dummy Host Bridge
40:08.1 PCI bridge: Advanced Micro Devices, Inc. [AMD] Family 17h
(Models 00h-0fh) Internal PCIe GPP Bridge 0 to Bus B
41:00.0 VGA compatible controller: NVIDIA Corporation Device 1f02 (rev
a1)
41:00.1 Audio device: NVIDIA Corporation Device 10f9 (rev a1)
41:00.2 USB controller: NVIDIA Corporation Device 1ada (rev a1)
41:00.3 Serial bus controller [0c80]: NVIDIA Corporation Device 1adb
(rev a1)
42:00.0 Non-Essential Instrumentation [1300]: Advanced Micro Devices,
Inc. [AMD] Device 145a
42:00.2 Encryption controller: Advanced Micro Devices, Inc. [AMD]
Family 17h (Models 00h-0fh) Platform Security Processor
42:00.3 USB controller: Advanced Micro Devices, Inc. [AMD] USB 3.0
Host controller
43:00.0 Non-Essential Instrumentation [1300]: Advanced Micro Devices,
Inc. [AMD] Device 1455
43:00.2 SATA controller: Advanced Micro Devices, Inc. [AMD] FCH SATA
Controller [AHCI mode] (rev 51)
```

****
For PYTHON FIM Codes (Figures 2-4, S1-S10)

Codes Tested on multiple devices:

DEVICE 1 (MacBook Pro Laptop with M1 chip)

Software:

System Software Overview:

System Version: macOS 13.3.1 (22E261)
Kernel Version: Darwin 22.4.0

```
      Boot Volume: Macintosh HD
      Boot Mode: Normal
      Secure Virtual Memory: Enabled
      System Integrity Protection: Enabled
      Time since boot: 3 days, 6 hours, 52 minutes

Hardware:

    Hardware Overview:

      Model Name: MacBook Pro
      Model Identifier: MacBookPro17,1
      Model Number: Z11C000E4LL/A
      Chip: Apple M1
      Total Number of Cores: 8 (4 performance and 4 efficiency)
      Memory: 16 GB
      System Firmware Version: 8422.100.650
      OS Loader Version: 8422.100.650


DEVICE 2 (Rocky Linux Cluster):

Architecture:          x86_64
CPU op-mode(s):        32-bit, 64-bit
Byte Order:            Little Endian
CPU(s):                12
On-line CPU(s) list: 0-11
Thread(s) per core:  1
Core(s) per socket:  6
Socket(s):             2
NUMA node(s):          2
Vendor ID:             GenuineIntel
CPU family:            6
Model:                 63
Model name:            Intel(R) Xeon(R) CPU E5-2620 v3 @ 2.40GHz
Stepping:              2
CPU MHz:               3200.000
CPU max MHz:           3200.0000
CPU min MHz:           1200.0000
BogoMIPS:              4800.45
L1d cache:             32K
L1i cache:             32K
L2 cache:              256K
L3 cache:              15360K
NUMA node0 CPU(s):   0-5
NUMA node1 CPU(s):   6-11

NAME="Rocky Linux"
VERSION="8.7 (Green Obsidian)"
ID="rocky"
```

ID_LIKE="rhel centos fedora"
VERSION_ID="8.7"
PLATFORM_ID="platform:el8"
PRETTY_NAME="Rocky Linux 8.7 (Green Obsidian)"
ANSI_COLOR="0;32"
LOGO="fedora-logo-icon"
CPE_NAME="cpe:/o:rocky:rocky:8:GA"
HOME_URL="https://rockylinux.org/"
BUG_REPORT_URL="https://bugs.rockylinux.org/"
ROCKY_SUPPORT_PRODUCT="Rocky-Linux-8"
ROCKY_SUPPORT_PRODUCT_VERSION="8.7"
REDHAT_SUPPORT_PRODUCT="Rocky Linux"
REDHAT_SUPPORT_PRODUCT_VERSION="8.7"

****
For MATLAB Codes (Figures 5-8, S11, S12)

Codes Tested on multiple devices:

DEVICE 1 (MacBook Pro Laptop):
Hardware:

```
    Hardware Overview:

      Model Name: MacBook Pro
      Model Identifier: MacBookPro14,3
      Processor Name: Quad-Core Intel Core i7
      Processor Speed: 2.9 GHz
      Number of Processors: 1
      Total Number of Cores: 4
      L2 Cache (per Core): 256 KB
      L3 Cache: 8 MB
      Hyper-Threading Technology: Enabled
      Memory: 16 GB
      System Firmware Version: 499.40.2.0.0
      OS Loader Version: 540.120.3~22
      SMC Version (system): 2.45f5
```

Software:

```
    System Software Overview:

      System Version: macOS 12.6.3 (21G419)
      Kernel Version: Darwin 21.6.0
      Boot Mode: Normal
      Secure Virtual Memory: Enabled
      System Integrity Protection: Enabled
```

DEVICE 2 (Rocky Linux Cluster):

```
Architecture:        x86_64
CPU op-mode(s):      32-bit, 64-bit
Byte Order:          Little Endian
CPU(s):              12
On-line CPU(s) list: 0-11
Thread(s) per core:  1
Core(s) per socket:  6
Socket(s):           2
NUMA node(s):        2
Vendor ID:           GenuineIntel
CPU family:          6
Model:               63
Model name:          Intel(R) Xeon(R) CPU E5-2620 v3 @ 2.40GHz
Stepping:            2
CPU MHz:             3200.000
CPU max MHz:         3200.0000
CPU min MHz:         1200.0000
BogoMIPS:            4800.45
L1d cache:           32K
L1i cache:           32K
L2 cache:            256K
L3 cache:            15360K
NUMA node0 CPU(s):   0-5
NUMA node1 CPU(s):   6-11

NAME="Rocky Linux"
VERSION="8.7 (Green Obsidian)"
ID="rocky"
ID_LIKE="rhel centos fedora"
VERSION_ID="8.7"
PLATFORM_ID="platform:el8"
PRETTY_NAME="Rocky Linux 8.7 (Green Obsidian)"
ANSI_COLOR="0;32"
LOGO="fedora-logo-icon"
CPE_NAME="cpe:/o:rocky:rocky:8:GA"
HOME_URL="https://rockylinux.org/"
BUG_REPORT_URL="https://bugs.rockylinux.org/"
ROCKY_SUPPORT_PRODUCT="Rocky-Linux-8"
ROCKY_SUPPORT_PRODUCT_VERSION="8.7"
REDHAT_SUPPORT_PRODUCT="Rocky Linux"
REDHAT_SUPPORT_PRODUCT_VERSION="8.7"
```

MATLAB Scripts have been tested on multiple versions.

MATLAB Scripts tested on Versions R2021b, R2022b, and R2023a. Version Information provided below:


```
MATLAB Version: 9.11.0.2022996 (R2021b) Update 4
MATLAB License Number: 40638290
Operating System: macOS  Version: 12.6.3 Build: 21G419
Java Version: Java 1.8.0_202-b08 with Oracle Corporation Java
HotSpot(TM) 64-Bit Server VM mixed mode
----------------------------------------------------------------------
-------------------------------
MATLAB                                                Version 9.11
(R2021b)
Simulink                                              Version 10.4
(R2021b)
5G Toolbox                                            Version 2.3
(R2021b)
AUTOSAR Blockset                                      Version 2.5
(R2021b)
Aerospace Blockset                                    Version 5.1
(R2021b)
Aerospace Toolbox                                     Version 4.1
(R2021b)
Antenna Toolbox                                       Version 5.1
(R2021b)
Audio Toolbox                                         Version 3.1
(R2021b)
Automated Driving Toolbox                             Version 3.4
(R2021b)
Bioinformatics Toolbox                                Version 4.15.2
(R2021b)
Communications Toolbox                                Version 7.6
(R2021b)
Computer Vision Toolbox                               Version 10.1
(R2021b)
Control System Toolbox                                Version 10.11
(R2021b)
Curve Fitting Toolbox                                 Version 3.6
(R2021b)
DDS Blockset                                          Version 1.1
(R2021b)
DSP System Toolbox                                    Version 9.13
(R2021b)
Database Toolbox                                      Version 10.2
(R2021b)
Datafeed Toolbox                                      Version 6.1
(R2021b)
Deep Learning Toolbox                                 Version 14.3
(R2021b)
Econometrics Toolbox                                  Version 5.7
(R2021b)
Embedded Coder                                        Version 7.7
(R2021b)
Filter Design HDL Coder                               Version 3.1.10
```

```
(R2021b)
Financial Instruments Toolbox                         Version 3.3
(R2021b)
Financial Toolbox                                     Version 6.2
(R2021b)
Fixed-Point Designer                                  Version 7.3
(R2021b)
Fuzzy Logic Toolbox                                   Version 2.8.2
(R2021b)
Global Optimization Toolbox                           Version 4.6
(R2021b)
HDL Coder                                             Version 3.19
(R2021b)
Image Acquisition Toolbox                             Version 6.5
(R2021b)
Image Processing Toolbox                              Version 11.4
(R2021b)
Instrument Control Toolbox                            Version 4.5
(R2021b)
LTE Toolbox                                           Version 3.6
(R2021b)
Lidar Toolbox                                         Version 2.0
(R2021b)
MATLAB Coder                                          Version 5.3
(R2021b)
MATLAB Compiler                                       Version 8.3
(R2021b)
MATLAB Compiler SDK                                   Version 6.11
(R2021b)
MATLAB Report Generator                               Version 5.11
(R2021b)
Mapping Toolbox                                       Version 5.2
(R2021b)
Mixed-Signal Blockset                                 Version 2.1
(R2021b)
Model Predictive Control Toolbox                      Version 7.2
(R2021b)
Motor Control Blockset                                Version 1.3
(R2021b)
Navigation Toolbox                                    Version 2.1
(R2021b)
Optimization Toolbox                                  Version 9.2
(R2021b)
Parallel Computing Toolbox                            Version 7.5
(R2021b)
Partial Differential Equation Toolbox                 Version 3.7
(R2021b)
Phased Array System Toolbox                           Version 4.6
(R2021b)
Powertrain Blockset                                   Version 1.10
```

(R2021b)
Predictive Maintenance Toolbox Version 2.4
(R2021b)
RF Blockset Version 8.2
(R2021b)
RF PCB Toolbox Version 1.0
(R2021b)
RF Toolbox Version 4.2
(R2021b)
ROS Toolbox Version 1.4
(R2021b)
Radar Toolbox Version 1.1
(R2021b)
Reinforcement Learning Toolbox Version 2.1
(R2021b)
Risk Management Toolbox Version 1.10
(R2021b)
Robotics System Toolbox Version 3.4
(R2021b)
Robust Control Toolbox Version 6.11
(R2021b)
Satellite Communications Toolbox Version 1.1
(R2021b)
Sensor Fusion and Tracking Toolbox Version 2.2
(R2021b)
SerDes Toolbox Version 2.2
(R2021b)
Signal Processing Toolbox Version 8.7
(R2021b)
SimBiology Version 6.2
(R2021b)
SimEvents Version 5.11
(R2021b)
Simscape Version 5.2
(R2021b)
Simscape Driveline Version 3.4
(R2021b)
Simscape Electrical Version 7.6
(R2021b)
Simscape Fluids Version 3.3
(R2021b)
Simscape Multibody Version 7.4
(R2021b)
Simulink 3D Animation Version 9.3
(R2021b)
Simulink Check Version 5.2
(R2021b)
Simulink Coder Version 9.6
(R2021b)
Simulink Compiler Version 1.3

```
(R2021b)
Simulink Control Design                                    Version 6.0
(R2021b)
Simulink Coverage                                          Version 5.3
(R2021b)
Simulink Design Optimization                               Version 3.10
(R2021b)
Simulink Design Verifier                                   Version 4.6
(R2021b)
Simulink Desktop Real-Time                                 Version 5.13
(R2021b)
Simulink PLC Coder                                         Version 3.5
(R2021b)
Simulink Report Generator                                  Version 5.11
(R2021b)
Simulink Requirements                                      Version 1.8
(R2021b)
Simulink Test                                              Version 3.5
(R2021b)
Stateflow                                                  Version 10.5
(R2021b)
Statistics and Machine Learning Toolbox                    Version 12.2
(R2021b)
Symbolic Math Toolbox                                      Version 9.0
(R2021b)
System Composer                                            Version 2.1
(R2021b)
System Identification Toolbox                              Version 9.15
(R2021b)
Tensor Toolbox (Sandia Natl Labs & MathSci.ai)             Version 3.4
(R2022c)
Text Analytics Toolbox                                     Version 1.8
(R2021b)
UAV Toolbox                                                Version 1.2
(R2021b)
Vehicle Dynamics Blockset                                  Version 1.7
(R2021b)
WLAN Toolbox                                               Version 3.3
(R2021b)
Wavelet Toolbox                                            Version 6.0
(R2021b)
Wireless HDL Toolbox                                       Version 2.3
(R2021b)

----------------------------------------------------------------------
-------------------------------
MATLAB Version: 9.14.0.2239454 (R2023a) Update 1
MATLAB License Number: 40638290
Operating System: macOS  Version: 12.6.3 Build: 21G419
Java Version: Java 1.8.0_202-b08 with Oracle Corporation Java
```

```
HotSpot(TM) 64-Bit Server VM mixed mode
-----------------------------------------------------------------------------------------------------
MATLAB                                                     Version 9.14
(R2023a)
Simulink                                                   Version 10.7
(R2023a)
Audio Toolbox                                              Version 3.4
(R2023a)
Bioinformatics Toolbox                                     Version 4.17
(R2023a)
Communications Toolbox                                     Version 8.0
(R2023a)
Computer Vision Toolbox                                    Version 10.4
(R2023a)
Control System Toolbox                                     Version 10.13
(R2023a)
Curve Fitting Toolbox                                      Version 3.9
(R2023a)
DSP HDL Toolbox                                            Version 1.2
(R2023a)
DSP System Toolbox                                         Version 9.16
(R2023a)
Database Toolbox                                           Version 11.0
(R2023a)
Deep Learning Toolbox                                      Version 14.6
(R2023a)
Econometrics Toolbox                                       Version 6.2
(R2023a)
Embedded Coder                                             Version 7.10
(R2023a)
Filter Design HDL Coder                                    Version 3.1.13
(R2023a)
Financial Toolbox                                          Version 6.5
(R2023a)
Fixed-Point Designer                                       Version 7.6
(R2023a)
Fuzzy Logic Toolbox                                        Version 3.1
(R2023a)
Global Optimization Toolbox                                Version 4.8.1
(R2023a)
HDL Coder                                                  Version 4.1
(R2023a)
Image Acquisition Toolbox                                  Version 6.7.1
(R2023a)
Image Processing Toolbox                                   Version 11.7
(R2023a)
MATLAB Coder                                               Version 5.6
(R2023a)
MATLAB Compiler                                            Version 8.6
```

(R2023a)
MATLAB Compiler SDK   Version 7.2
(R2023a)
MATLAB Report Generator   Version 5.14
(R2023a)
MATLAB Test   Version 1.0
(R2023a)
Mapping Toolbox   Version 5.5
(R2023a)
Medical Imaging Toolbox   Version 1.1
(R2023a)
Model Predictive Control Toolbox   Version 8.1
(R2023a)
Motor Control Blockset   Version 2.0
(R2023a)
Optimization Toolbox   Version 9.5
(R2023a)
Parallel Computing Toolbox   Version 7.8
(R2023a)
Partial Differential Equation Toolbox   Version 3.10
(R2023a)
Phased Array System Toolbox   Version 5.0
(R2023a)
Powertrain Blockset   Version 2.0
(R2023a)
Predictive Maintenance Toolbox   Version 2.7
(R2023a)
RF Blockset   Version 8.5
(R2023a)
RF PCB Toolbox   Version 1.3
(R2023a)
RF Toolbox   Version 4.5
(R2023a)
ROS Toolbox   Version 2.0
(R2023a)
Radar Toolbox   Version 1.4
(R2023a)
Reinforcement Learning Toolbox   Version 2.4
(R2023a)
Requirements Toolbox   Version 2.2
(R2023a)
Risk Management Toolbox   Version 2.2
(R2023a)
Robotics System Toolbox   Version 4.2
(R2023a)
Robust Control Toolbox   Version 6.11.3
(R2023a)
Satellite Communications Toolbox   Version 1.4
(R2023a)
Sensor Fusion and Tracking Toolbox   Version 2.5

(R2023a)
SerDes Toolbox   Version 3.0
(R2023a)
Signal Processing Toolbox   Version 9.2
(R2023a)
SimBiology   Version 6.4.1
(R2023a)
SimEvents   Version 5.14
(R2023a)
Simscape   Version 5.5
(R2023a)
Simscape Battery   Version 1.1
(R2023a)
Simscape Driveline   Version 3.7
(R2023a)
Simscape Electrical   Version 7.9
(R2023a)
Simscape Fluids   Version 3.6
(R2023a)
Simscape Multibody   Version 7.7
(R2023a)
Simulink 3D Animation   Version 9.6
(R2023a)
Simulink Check   Version 6.2
(R2023a)
Simulink Coder   Version 9.9
(R2023a)
Simulink Compiler   Version 1.6
(R2023a)
Simulink Control Design   Version 7.0
(R2023a)
Simulink Coverage   Version 5.6
(R2023a)
Simulink Design Optimization   Version 3.13
(R2023a)
Simulink Design Verifier   Version 4.9
(R2023a)
Simulink Desktop Real-Time   Version 5.16
(R2023a)
Simulink PLC Coder   Version 3.8
(R2023a)
Simulink Report Generator   Version 5.14
(R2023a)
Simulink Test   Version 3.8
(R2023a)
Stateflow   Version 10.8
(R2023a)
Statistics and Machine Learning Toolbox   Version 12.5
(R2023a)
Symbolic Math Toolbox   Version 9.3

```
(R2023a)
System Composer                                      Version 2.4
(R2023a)
System Identification Toolbox                        Version 10.1
(R2023a)
Tensor Toolbox (Sandia Natl Labs & MathSci.ai)       Version 3.4
(R2022c)
Text Analytics Toolbox                               Version 1.10
(R2023a)
UAV Toolbox                                          Version 1.5
(R2023a)
Vehicle Dynamics Blockset                            Version 2.0
(R2023a)
WLAN Toolbox                                         Version 3.6
(R2023a)
Wavelet Toolbox                                      Version 6.3
(R2023a)
Wireless HDL Toolbox                                 Version 2.6
(R2023a)

----------------------------------------------------------------------
-------------------------------
MATLAB Version: 9.13.0.2080170 (R2022b) Update 1
MATLAB License Number: 40638290
Operating System: Linux 4.18.0-348.12.2.el8_5.x86_64 #1 SMP Wed Jan 19
17:53:40 UTC 2022 x86_64
Java Version: Java 1.8.0_202-b08 with Oracle Corporation Java
HotSpot(TM) 64-Bit Server VM mixed mode
----------------------------------------------------------------------
-------------------------------
MATLAB                                               Version 9.13
(R2022b)
Simulink                                             Version 10.6
(R2022b)
5G Toolbox                                           Version 2.5
(R2022b)
AUTOSAR Blockset                                     Version 3.0
(R2022b)
Aerospace Blockset                                   Version 5.3
(R2022b)
Aerospace Toolbox                                    Version 4.3
(R2022b)
Antenna Toolbox                                      Version 5.3
(R2022b)
Audio Toolbox                                        Version 3.3
(R2022b)
Automated Driving Toolbox                            Version 3.6
(R2022b)
Bioinformatics Toolbox                               Version 4.16.1
(R2022b)
```

Bluetooth Toolbox (R2022b)	Version 1.1
Communications Toolbox (R2022b)	Version 7.8
Computer Vision Toolbox (R2022b)	Version 10.3
Control System Toolbox (R2022b)	Version 10.12
Curve Fitting Toolbox (R2022b)	Version 3.8
DDS Blockset (R2022b)	Version 1.3
DSP HDL Toolbox (R2022b)	Version 1.1
DSP System Toolbox (R2022b)	Version 9.15
Database Toolbox (R2022b)	Version 10.4
Datafeed Toolbox (R2022b)	Version 6.3
Deep Learning HDL Toolbox (R2022b)	Version 1.4
Deep Learning Toolbox (R2022b)	Version 14.5
Econometrics Toolbox (R2022b)	Version 6.1
Embedded Coder (R2022b)	Version 7.9
Filter Design HDL Coder (R2022b)	Version 3.1.12
Financial Instruments Toolbox (R2022b)	Version 3.5
Financial Toolbox (R2022b)	Version 6.4
Fixed-Point Designer (R2022b)	Version 7.5
Fuzzy Logic Toolbox (R2022b)	Version 3.0
GPU Coder (R2022b)	Version 2.4
Global Optimization Toolbox (R2022b)	Version 4.8
HDL Coder (R2022b)	Version 4.0
HDL Verifier (R2022b)	Version 7.0
Image Acquisition Toolbox (R2022b)	Version 6.7
Image Processing Toolbox (R2022b)	Version 11.6

Industrial Communication Toolbox (R2022b)	Version 6.1
Instrument Control Toolbox (R2022b)	Version 4.7
LTE Toolbox (R2022b)	Version 3.8
Lidar Toolbox (R2022b)	Version 2.2
MATLAB Coder (R2022b)	Version 5.5
MATLAB Compiler (R2022b)	Version 8.5
MATLAB Compiler SDK (R2022b)	Version 7.1
MATLAB Report Generator (R2022b)	Version 5.13
Mapping Toolbox (R2022b)	Version 5.4
Medical Imaging Toolbox (R2022b)	Version 1.0
Mixed-Signal Blockset (R2022b)	Version 2.3
Model Predictive Control Toolbox (R2022b)	Version 8.0
Motor Control Blockset (R2022b)	Version 1.5
Navigation Toolbox (R2022b)	Version 2.3
Optimization Toolbox (R2022b)	Version 9.4
Parallel Computing Toolbox (R2022b)	Version 7.7
Partial Differential Equation Toolbox (R2022b)	Version 3.9
Phased Array System Toolbox (R2022b)	Version 4.8
Powertrain Blockset (R2022b)	Version 1.12
Predictive Maintenance Toolbox (R2022b)	Version 2.6
RF Blockset (R2022b)	Version 8.4
RF PCB Toolbox (R2022b)	Version 1.2
RF Toolbox (R2022b)	Version 4.4
ROS Toolbox (R2022b)	Version 1.6
Radar Toolbox (R2022b)	Version 1.3

Reinforcement Learning Toolbox (R2022b)	Version 2.3
Requirements Toolbox (R2022b)	Version 2.1
Risk Management Toolbox (R2022b)	Version 2.1
Robotics System Toolbox (R2022b)	Version 4.1
Robust Control Toolbox (R2022b)	Version 6.11.2
Satellite Communications Toolbox (R2022b)	Version 1.3
Sensor Fusion and Tracking Toolbox (R2022b)	Version 2.4
SerDes Toolbox (R2022b)	Version 2.4
Signal Integrity Toolbox (R2022b)	Version 1.2
Signal Processing Toolbox (R2022b)	Version 9.1
SimBiology (R2022b)	Version 6.4
SimEvents (R2022b)	Version 5.13
Simscape (R2022b)	Version 5.4
Simscape Battery (R2022b)	Version 1.0
Simscape Driveline (R2022b)	Version 3.6
Simscape Electrical (R2022b)	Version 7.8
Simscape Fluids (R2022b)	Version 3.5
Simscape Multibody (R2022b)	Version 7.6
Simulink 3D Animation (R2022b)	Version 9.5
Simulink Check (R2022b)	Version 6.1
Simulink Code Inspector (R2022b)	Version 4.2
Simulink Coder (R2022b)	Version 9.8
Simulink Compiler (R2022b)	Version 1.5
Simulink Control Design (R2022b)	Version 6.2
Simulink Coverage (R2022b)	Version 5.5

```
Simulink Design Optimization                              Version 3.12
(R2022b)
Simulink Design Verifier                                  Version 4.8
(R2022b)
Simulink PLC Coder                                        Version 3.7
(R2022b)
Simulink Real-Time                                        Version 8.1
(R2022b)
Simulink Report Generator                                 Version 5.13
(R2022b)
Simulink Test                                             Version 3.7
(R2022b)
SoC Blockset                                              Version 1.7
(R2022b)
Stateflow                                                 Version 10.7
(R2022b)
Statistics and Machine Learning Toolbox                   Version 12.4
(R2022b)
Symbolic Math Toolbox                                     Version 9.2
(R2022b)
System Composer                                           Version 2.3
(R2022b)
System Identification Toolbox                             Version 10.0
(R2022b)
Text Analytics Toolbox                                    Version 1.9
(R2022b)
UAV Toolbox                                               Version 1.4
(R2022b)
Vehicle Dynamics Blockset                                 Version 1.9
(R2022b)
Vehicle Network Toolbox                                   Version 5.3
(R2022b)
Vision HDL Toolbox                                        Version 2.6
(R2022b)
WLAN Toolbox                                              Version 3.5
(R2022b)
Wavelet Toolbox                                           Version 6.2
(R2022b)
Wireless HDL Toolbox                                      Version 2.5
(R2022b)
Wireless Testbench                                        Version 1.1
(R2022b)
```
